# TRAIL and IP-10 dynamics in pregnant women post COVID-19 vaccination: associations with neutralizing antibody potency

**DOI:** 10.3389/fcimb.2024.1358967

**Published:** 2024-03-20

**Authors:** Wei-Chun Chen, Shu-Yu Hu, Chao-Min Cheng, Ching-Fen Shen, Hui-Yu Chuang, Chin-Ru Ker, Der-Ji Sun, Ching-Ju Shen

**Affiliations:** ^1^ Institute of Biomedical Engineering, National Tsing Hua University, Hsinchu, Taiwan; ^2^ Division of Gynecologic Oncology, Department of Obstetrics and Gynecology, Chang Gung Memorial Hospital at Linkou, College of Medicine, Chang Gung University, Taoyuan, Taiwan; ^3^ Department of Obstetrics and Gynecology, New Taipei City Municipal Tucheng Hospital, New Taipei City, Taiwan; ^4^ International Intercollegiate Ph.D. Program, National Tsing Hua University, Hsinchu, Taiwan; ^5^ School of Traditional Chinese Medicine, Chang Gung University, Taoyuan, Taiwan; ^6^ Department of Pediatrics, National Cheng Kung University Hospital, College of Medicine, National Cheng Kung University, Tainan, Taiwan; ^7^ Department of Obstetrics and Gynecology, Kaohsiung Medical University Hospital, Kaohsiung Medical University, Kaohsiung, Taiwan; ^8^ Graduate Institute of Clinical Medicine, College of Medicine, Kaohsiung Medical University, Kaohsiung, Taiwan; ^9^ Department of Obstetrics and Gynecology, Pojen Hospital, Kaohsiung, Taiwan

**Keywords:** TNF-related apoptosis-inducing ligand, TRAIL, interferon gamma-induced protein 10, IP-10, COVID-19 vaccine, neutralizing antibody, nab

## Abstract

**Introduction:**

The aim of this study is to investigate changes in TNF-related apoptosis-inducing ligand (TRAIL) and gamma interferon-induced protein 10 (IP-10) after COVID-19 vaccination in pregnant women and to explore their association with neutralizing antibody (Nab) inhibition.

**Methods:**

The study evaluated 93 pregnant women who had previously received two (n=21), three (n=55) or four (n=17) doses of COVID-19 vaccine. Also we evaluated maternal blood samples that were collected during childbirth. The levels of TRAIL, IP-10 and Nab inhibition were measured using enzyme-linked immunosorbent assays (ELISA).

**Results and discussion:**

Our study revealed four-dose group resulted in lower TRAIL levels when compared to the two-dose and three-dose groups (4.78 vs. 16.07 vs. 21.61 pg/ml, p = 0.014). The two-dose group had reduced IP-10 levels than the three-dose cohort (111.49 vs. 147.89 pg/ml, p=0.013), with no significant variation compared to the four-dose group. In addition, the four-dose group showed stronger Nab inhibition against specific strains (BA.2 and BA.5) than the three-dose group. A positive correlation was observed between TRAIL and IP-10 in the two-dose group, while this relationship was not found in other dose groups or between TRAIL/IP-10 and Nab inhibition. As the doses of the COVID-19 vaccine increase, the levels of TRAIL and IP-10 generally increase, only by the fourth dose, the group previously vaccinated with AZD1222 showed lower TRAIL but higher IP-10. Despite these changes, more doses of the vaccine consistently reinforced Nab inhibition, apparently without any relation to TRAIL and IP-10 levels. The variation may indicate the induction of immunological memory in vaccinated mothers, which justifies further research in the future.

## 1 Introduction

Since the emergence of the COVID-19 pandemic in 2019, the disease caused by the SARS-CoV-2 virus has turned into a global health crisis with a remarkable transmission rate (Borges Do Nascimento et al., 2021). Vaccination has been crucial in curbing its spread and reducing the incidence of severe cases, particularly among vulnerable groups such as healthcare professionals and pregnant women (Polack et al., 2020; Butt et al., 2021). The literature indicates that pregnant women who contract COVID-19 have an increased risk of severe complications such as preeclampsia, preterm birth, and emergency Cesarean deliveries, with 18% experiencing severe illness or critical morbidity (Pettirosso et al., 2020; Gurol-Urganci et al., 2021). Furthermore, neonates of infected mothers face extended hospital stays, necessitating specialized care, and an increased likelihood of adverse outcomes (Zimmermann and Curtis, 2020). Various types of COVID-19 vaccines, including inactivated vaccines, nucleic acid-based vaccines (mRNA and DNA vaccines), protein-based vaccines, and adenovirus vector vaccines are available (Ghasemiyeh et al., 2021; Li et al., 2021; Nagy and Alhatlani, 2021). Vaccination against COVID-19 for pregnant women allow mothers to produce and transfer protective antibodies to their fetus (Shen et al., 2022). Specifically, the transplacental transmission of SARS-CoV-2 neutralizing antibodies (Nabs) has shown potential in protecting fetuses and neonates (Shen et al., 2022; Chen et al., 2022a), and this protection can also be observed for newly-evolved SARS-CoV-2 variants including Omicron variants (Chen et al., 2022b; Munoz et al., 2023). According to current research, COVID-19 vaccination in pregnant women does not increase the risk of any adverse pregnancy, maternal, or neonatal outcomes. This includes risks of spontaneous abortion, stillbirth, congenital anomalies, preterm birth, neonatal intensive care unit (NICU) admission, gestational diabetes, hypertensive disorders, and others, indicating that COVID-19 vaccination during pregnancy is safe (Fleming-Dutra et al., 2023).

Determining the severity of illness in infected COVID-19 patients is crucial. Several biomarkers, such as TNF-related apoptosis-inducing ligand (TRAIL), interferon gamma-induced protein 10 (IP-10 or CXCL10), and C-related protein (CRP), have been identified as indicators for assessing the severity of COVID-19 (Tegethoff et al., 2022). TRAIL, a member of the tumor necrosis factor (TNF) family, can induce apoptosis in cells through an extrinsic pathway by binding to death receptors (DR) (Condotta et al., 2013). Apoptosis can occur in immune cells, virus-infected cells, or tumor cells (Gyurkovska and Ivanovska, 2016). Hence, TRAIL plays a dual role, encompassing both immune-suppressive and immune-stimulatory functions (Ishikawa et al., 2005). Additionally, IP-10 is a chemokine released in response to inflammation. Inflammatory reactions can be generated when the body encounters an infection, and leukocytes as well as neutrophils can release IP-10 under the influence of IFN-g, thereby activating and recruiting B cells, T cells, and NK cells to combat foreign pathogens (Singh et al., 2008; Lu et al., 2011; Bergamaschi et al., 2021). Consequently, IP-10 levels increase when the body is infected with a virus. Current research also indicates that the levels of IP-10 may be affected by COVID-19 vaccine. After the first dose of COVID-19 vaccine, an increase in IP-10 is observed (Huang et al., 2005; Sobolev et al., 2016; Dunning et al., 2018). The levels of IP-10 can rise after the second dose (Bergamaschi et al., 2021). However, no literature has yet discussed the association between TRAIL and the COVID-19 vaccine.

In pregnant women diagnosed with COVID-19, elevated levels of IP-10 have been observed (Rosen et al., 2022). Additionally, the increased IP-10 level in both the diagnosed mother and her fetus have potential implications for the long-term health of the fetus (Taglauer et al., 2022). However, no literature has addressed the association between TRAIL and pregnant women diagnosed with COVID-19. Moreover, there is a lack of research examining the correlation between TRAIL or IP-10 levels and pregnant women post-COVID-19 vaccination. Therefore, our study aims to investigate the relationship between TRAIL and IP-10 in pregnant women who have received a COVID-19 vaccine. Concurrently, the association of neutralizing antibody inhibition for SARS-CoV-2 among mothers and the neonates will be also explored.

## 2 Materials and methods

### 2.1 Participants collection

The current study was performed at Kaohsiung Medical University Hospital and included only patients with singleton pregnancies. All participants were aged 20 or above. Additionally, any subjects that experienced preterm labor, displayed symptoms associated with COVID-19, or demonstrated prior medical history indicating the need for immunosuppressant treatments were purposefully excluded from this investigation. The study was conducted after receiving approval from the local institutional review board (IRB); designated IRB number: KMUHIRB-SV(II)-20210087.

In our study, all participants were previous recipients of 2 to 4 doses of COVID-19 vaccine. Among those receiving 4 doses, the 4^th^ dose was the Moderna COVID-19 bivalent (SPIKEVAX Bivalent Original/Omicron BA.1 or BA.4/5) vaccine. Those subjects that received two doses of COVID-19 vaccine received both vaccines during their pregnancy, and they typically received the Pfizer BioNTech (BNT162b2) COVID-19 vaccine or the Spikevax (elasomeran) COVID-19 vaccine (previous called the mRNA-1273 Moderna vaccine). By contrast, participants receiving 3 or 4 doses had their final shot during their pregnancy, but their preceding doses might have been administered before conception. These subjects received the Oxford/AstraZeneca ChAdOx1 nCoV-19 (AZD1222) vaccine, mRNA-1273 Moderna vaccine, or BNT162b2 vaccine.

Additionally, participants in our study were allowed to receive standard vaccinations during their antenatal period that included the tetanus toxoid, reduced diphtheria toxoid, and acellular pertussis (Tdap) vaccines (Adacel, Sanofi Pasteur, Toronto, Ontario, Canada) and the influenza (Flu) vaccine (AdimFlu-S, QIS, Adimmune Corporation, Taichung, Taiwan; FlucelvaxQuad, CSL Behring GmbH, Marburg, Germany; VAXIGRIP TETRA, Sanofi Pasteur, Val-de-Reuil, Cedex, France). No significant discomfort or morbidity was reported after vaccination during investigation.

### 2.2 Sample collection

All eligible pregnant women were admitted to our study on the day of delivery after obtaining permission via informed consent. Blood samples were collected from the pregnant mother and the neonatal umbilical cord post cord-clamping on the delivery day. These samples were then sent to the laboratory for further analysis. Concurrently, pertinent clinical data was extracted from the electronic medical record system for comprehensive statistical evaluation including maternal age, parity, body mass index (BMI), neonatal weight, baby’s gender, dates of COVID-19 vaccination and other vital parameters. All compiled data was used for our subsequent analysis.

### 2.3 Detection of TRAIL and IP-10 level in maternal blood samples via enzyme-linked immunosorbent assays

In our study, we analyzed two targeted biomarkers, TRAIL and IP-10, in the collected maternal blood samples. Frozen specimens were thawed at room temperature, and the concentrations of TRAIL and IP-10 were quantified using enzyme-linked immunosorbent assays (ELISAs). For this procedure, we followed the manufacturer’s protocols (R&D systems Cat. No. DTRL00/DIP100), and subsequent absorbance value data was obtained using a plate reader following ELISA process. A calculated standard curve was used for further transformation of the above values into sample biomarker concentrations.

### 2.4 Detection of neutralizing antibody inhibition for SARS-CoV-2 Omicron subtype BA.1, BA.2, and BA.5

Neutralizing antibody (Nab) inhibition was detected via a competitive ELISA test that included a spike protein receptor binding domain (SRBD) solution that could interact with both the Nab from our collected samples and the angiotensin-converting enzyme 2 (ACE2) that was pre-coated in the wells of 96-well microplates. Analysis was performed using a commercially available ELISA kit for different SARS-CoV-2 subvariants under the manufacturer’s protocols (Acro biosystems Cat. No. RAS-N056/RAS-N087/RAS-N107).

The procedure began with the introduction of the collected samples along with both positive and negative controls into 96-well microplates. We then added the solution comprising horseradish peroxidase (HRP)-conjugated SRBD tailored for the respective SARS-CoV-2 variants. These microplates underwent incubation in the dark for 1 hour at room temperature. After incubation, we discarded the supernatant, thoroughly washed the wells, and introduced an additional substrate solution. After a subsequently repeated 20-minute incubation in the dark, a stopping solution was added. At this point, the microplate wells displayed a noticeable colorimetric change from blue to yellow. We evaluated the intensity of this colorimetric alteration in each well using a microplate spectrophotometer (Molecular Devices, USA) to read the O.D. value absorbance at 450 nm, and the obtained O.D values were employed to calculated the Nab inhibition percentage using the following formula:


$$\text{Inhibition \% }=\left(\;1-\frac{OD450\;value\;of\;sample}{average\;OD450\;value\;of\;negative\;control}\right)*100\%$$

### 2.5 Statistics

We employed the chi-square test to compare proportionate differences among participants receiving different doses of the COVID-19 vaccine. Based on these proportions, participants were categorized into distinct subgroups. Subsequent analyses using analysis of variance (ANOVA) or the sample t-test were conducted to calculate the differences in TRAIL and IP-10 values between these subgroups. Furthermore, we explored the relationships between the levels of TRAIL and IP-10, as well as the correlation between Nab inhibition and both TRAIL and IP-10 among the cohorts receiving different COVID-19 vaccine doses by Spearman and Pearson correlation. All data processing and analyses were performed using SPSS Statistics (version 27, IBM, USA) and Microsoft Excel (Microsoft, Redmond, Washington, USA). Calculated data demonstrating p-values less than 0.05 were considered statistically significant. We also illustrated these statistical findings using GraphPad Prism software (GraphPad Software, San Diego, CA, USA).

## 3 Results

### 3.1 Participants characteristics

We analyzed data from 93 eligible pregnant women during the investigation period. Among the participants, 21 had received two doses of COVID-19 vaccine, 55 had received three doses, and 17 had received four doses. The related characteristics are tabulated in **Table 1**. The mean age for these women was between 33 and 35 years, and the mean BMI was around 25 and 28. Analyzing the interval from the last dose of COVID-19 vaccination to childbirth revealed that there was a uniform distribution of subjects across each of the intervals, i.e., 0-4 weeks, 5-8 weeks, and 9-12 weeks. For the three-dose cohorts, 34.5% had vaccination-to-childbirth intervals of 0-4 weeks, 38.2% had intervals of 5-8 weeks, and 27.3% had intervals of 9-12 weeks. Among the four-dose participants, 35.3% had intervals of both 0-4 weeks and 5-8 weeks, 11.8% had intervals of 9-12 weeks, and a notable 17.6% exceeded 12 weeks.

**Table 1:** Participants characteristics.

	2 doses (N=21)	3 doses (N=55)	4 doses (N=17)	p value
Mean age (years)	34.1 (26 – 41)	33.2 (22 – 45)	33.2 (25 – 43)	0.411
Mean BMI	28.03 (21 – 40.84)*	27.89 (20.45 – 38.58)	25.78 (20.29 – 33.82)	0.321
Mean delivery weeks	37.7 (34 – 40)*	38.6 (37 – 40)	38.9 (38 – 40)	0.226
Last interval 0-4 weeks 5-8 weeks 9-12 weeks > 12 weeks	7 (33.3) 7 (33.3) 7 (33.3) 0	19 (34.5) 21 (38.2) 15 (27.3) 0	6 (35.3) 6 (35.3) 2 (11.8) 3 (17.6)	0.018
AZ included Yes Not	0 21 (100)	19 (34.5) 36 (65.5)	11 (64.7) 6 (35.3)	0.001
Tdap/Flu Tdap only Flu only Tdap + Flu No Tdap/Flu	0 0 0 21 (100)	28 (50.9) 1 (1.8) 16 (29.1) 10 (18.2)	3 (17.6) 0 11 (64.7) 3 (17.6)	0.001
Mean neonatal BW (gm)	3002.3 (2425 – 3550)*	3121.5 (2045 – 3855)	3065.0 (2325 – 3645)	0.583
Neonatal gender Male Female	6 (28.6) 15 (71.4)	31 (56.4) 24 (43.6)	9 (52.9) 8 (47.1)	0.091

*1 participant had miss data.

BMI, body mass index; AZ, AZD1222 vaccines; Tdap, tetanus toxoid, reduced diphtheria toxoid, and acellular pertussis vaccines; Flu, influenza vaccine; BW, body weight.

Regarding previous vaccination history, significant differences in vaccination history were detected among participants in the three cohorts that received AZD1222 vaccination. No participants in the two-dose group received the AZD1222 vaccine. In the three-dose and four-dose groups, the figures stood at 34.5% and 64.7% respectively. Significant differences were also found regarding Tdap and Flu vaccines. No participants in the two-dose cohorts received them; however, 50.9% of those in the three-dose group received only the Tdap instead of Flu vaccines, and 64.7% in the four-dose cohorts received both vaccines. As for neonatal outcomes, no significant differences were detected among the three cohorts. The median fetal weight spanned from 2900 to 3200 grams. Female neonates comprised 71.4% in the two-dose group, and approximately 43.6% and 47.1% in the three-dose and four-dose cohorts respectively.

### 3.2 Levels of TRAIL, IP-10, and Nab inhibition in our cohorts

**Figure 1:**
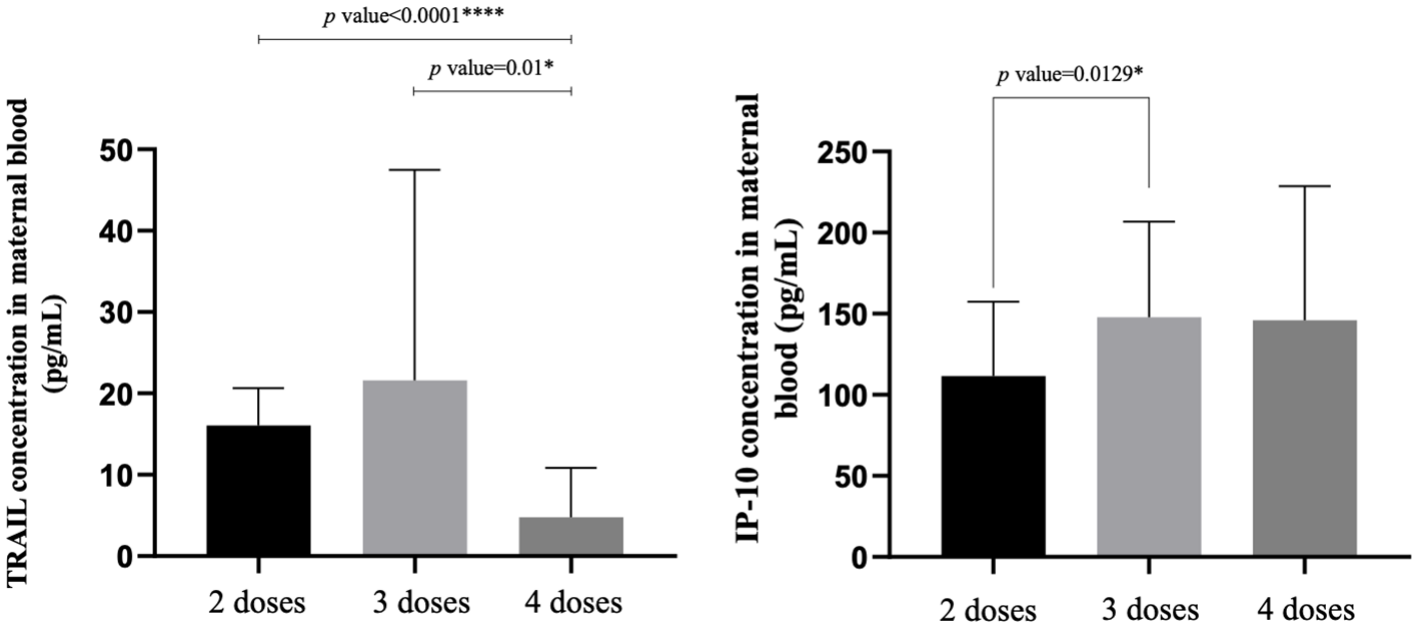
The maternal blood levels of TRAIL and IP-10 among participants receiving 2 doses, 3 doses, and 4 doses of COVID-19 vaccine. TNF-related apoptosis-inducing ligand, TRAIL; interferon gamma-induced protein 10, IP-10. *p-value < 0.05; ****p-value < 0.0001.

The TRAIL, IP-10, and Nab inhibition levels for the BA.1, BA.2, and BA.5 strains across cohorts with different dosages of vaccines are presented in **Table 2**, **Figure 1**, **Supplementary Figures S1**, and **S2**. Notably, the 4-dose group exhibited a significantly lower TRAIL value when compared to the 2-dose and 3-dose groups (4.78 pg/mL vs. 16.07 pg/mL vs. 21.61 pg/mL, respectively, p = 0.014). For IP-10 levels, the 2-dose group exhibited lower values than the 3-dose group (111.49 pg/mL vs. 147.89 pg/mL, p=0.013), while the 4-dose group levels did not differ significantly from those of the other two groups (145.92 pg/mL, p = 0.112 and 0.914 for 2-dose and 3-dose groups respectively). Regarding Nab inhibition rates, the 2-dose cohorts values for participants were not evaluated, but the 3-dose and 4-dose groups showed no significant difference in inhibition for the BA.1 strain (p=0.519). However, the 4-dose group demonstrated a significantly higher Nab inhibition rate for the BA.2 and BA.5 strains compared to the 3-dose group (BA.2: 56.32% vs. 25.28%, p<0.0001; BA.5: 48.38% vs. 17.27%, p<0.0001). Additionally, as showed in **Supplementary Figures S3** and **S4**, our analysis of the TRAIL and IP-10 responses across different dosage groups of Tdap and Flu vaccines reveals that despite variations in the administration of Tdap and Flu vaccines across groups, the profiles of TRAIL and IP-10 are fundamentally similar to those depicted in **Figure 1**. This suggests that the administration of Tdap and Flu vaccines does not significantly impact the expression patterns of TRAIL and IP-10.

**Table 2:** The levels of TRAIL, IP-10, and Nab inhibition rates to omicron type SARS-CoV-2 BA.1, BA.2, and BA.5 subvariants in maternal blood from participants receiving 2, 3, and 4 doses of COVID-19 vaccine.

	N	TRAIL (pg/mL)	IP-10 (pg/mL)	BA.1 (%)	BA.2 (%)	BA.5 (%)
2 doses 3 doses 4 doses p value	21 55 17	16.07 21.61 4.78 0.014	111.49 147.89 145.92 0.066	-^a^ 40.93^b^ 45.81 0.519	-^a^ 25.28^b^ 56.32 <0.0001	-^a^ 17.27^b^ 48.38 <0.0001
2 vs. 3 2 vs. 4 3 vs. 4		0.335 <0.0001 0.01	0.013 0.112 0.914			

a No available data for Nab concentration for the 2-doses group.

b 8 participants had no available data for Nab concentration.

TNF-related apoptosis-inducing ligand, TRAIL; interferon gamma-induced protein 10, IP-10; Nab, neutralizing antibody.

Moreover, within the 3-dose group, a longer interval between the third dose and childbirth corresponded to a significant decrease in Nab inhibition across BA.1, BA.2, and BA.5 (BA.1: p=0.0031 and 0.0006; BA.2: p=0.0144; BA.5: p=0.0145 and 0.0402), as illustrated in **Supplementary Figure S5**. Additionally, we also evaluated inhibition among participants that received both Tdap/Flu vaccines and compared those values to inhibition from participants who only received the Tdap vaccine within the 3-dose cohort. For the BA.1 and BA.5 strains, no significant difference in Nab inhibition was observed (BA.1: p=0.1627; BA.5: p=0.0599). However, for the BA.2 strain, those receiving both Tdap and Flu vaccines demonstrated greater Nab inhibition compared to those who received Tdap alone (p=0.0157). This data is visualized in **Supplementary Figure S6**.

### 3.3 Subgroup analysis of TRAIL and IP-10 levels comparing vaccine intervals and regimens

**Supplementary Table S1** presents a comparison of TRAIL and IP-10 levels among patients receiving different COVID-19 vaccine combinations. For cohorts that did not receive the AZD1222 vaccine, the 4-dose group demonstrated significantly lower TRAIL and IP-10 levels compared to both the 2-dose and 3-dose groups (TRAIL: 7.35 vs. 16.07 vs. 29.48 pg/mL, p=0.041; IP-10: 94.76 vs. 111.48 vs. 139.91 pg/mL, p=0.048). Among patients that did not receive the AZD1222 vaccine, there were no significant differences in TRAIL or IP-10 levels between the 3-dose and 4-dose groups (TRAIL: 6.68 vs. 3.38 pg/mL, p=0.122; IP-10: 162.99 vs. 173.83 pg/mL, p=0.631). Within individual vaccine dose groups, participants in the 3-dose group (and participants overall) exhibited higher TRAIL values when they had not been vaccinated with AZD1222 (3-dose: 29.48 vs. 6.68 pg/mL, p=0.013; overall: 22.90 vs. 5.47 pg/mL, p=0.001). For IP-10, a significant difference was observed in the overall participant group, i.e., those who received the AZD1222 vaccine showed significantly higher values than those who did not (166.97 vs. 126.12 pg/mL, p=0.0028).

**Figure 2:**
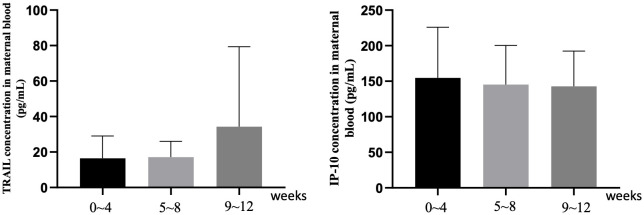
The maternal blood levels of TRAIL and IP-10 among participants with different intervals between last COIVD-19 vaccine and childbirth. TNF-related apoptosis-inducing ligand, TRAIL; interferon gamma-induced protein 10, IP-10.

**Supplementary Table S2** and **Figure 2** focus on the influence of the interval length between the final vaccine dose and childbirth on TRAIL and IP-10 levels. For TRAIL, there was no significant difference between different dosage groups for intervals of 0-4 weeks and 9-12 weeks (p=0.097 and 0.340). However, during the 5-8 week interval, the 4-dose group demonstrated significantly lower TRAIL levels compared to the 2-dose and 3-dose groups (4.68 vs. 15.79 vs. 17.15 pg/mL, p=0.01). The interval length did not significantly affect IP-10 levels across all groups. Additionally, within individual vaccine dose group, there remained no significant differences based on the interval from vaccination to childbirth.

### 3.4 Subgroup analysis of TRAIL and IP-10 levels in maternal factors

The influence of maternal factors, including maternal age, BMI, and Tdap/Flu vaccine administration on TRAIL and IP-10 levels is elaborated in **Supplementary Tables S3**, **S4**, and **S5** respectively. Among the maternal age group of 35-40 years, variation in TRAIL levels were evident among different dosage groups (p=0.040). For cohorts aged under 30 years, significant differences in IP-10 level were detected among different dosage groups (p=0.049), but there were no significant differences observed for other ages. Within each individual dosage cohorts, TRAIL levels within the 2-dose group exhibited significant differences based on maternal age (p=0.029), but other dosage groups showed no obvious differences. For IP-10, maternal age presented no significant impact among different dosage groups. Additionally, there were no differences in TRAIL and IP-10 levels observed based upon different maternal BMI.

Regarding the administration of Tdap or Flu vaccines during pregnancy, no significant differences were seen in IP-10 levels. However those who received both Tdap and Flu vaccines in the 4-dose group had significantly lower TRAIL levels compared to the 3-dose group (5.98 vs. 18.23 pg/mL, p=0.005). Similarly, those who did not receive Tdap or Flu vaccine displayed significantly lower TRAIL values in the 4-dose group compared to both the 2-dose and 3-dose groups (5.16 vs. 16.07 vs. 21.95 pg/mL, p=0.001). Nonetheless, within each individual dosage cohort, Tdap or Flu vaccination did not lead to any significant differential impacts on TRAIL or IP-10 values.

### 3.5 Subgroup analysis of TRAIL and IP-10 levels considering neonatal factors

The effect of neonatal factors, including neonatal body weight and gender, on TRAIL and IP-10 levels is listed in **Supplementary Tables S6** and **S7**. There were no significant differences among different dosage groups for both TRAIL and IP-10 levels regardless of neonatal body weight. Moreover, within individual dosage cohorts, variations in neonatal body weight had no significant effect on TRAIL or IP-10 levels. Regarding neonatal gender, male neonates in the 4-dose group exhibited significantly lower TRAIL levels compared to their counterparts in the 2-dose and 3-dose groups (5.30 vs. 17.77 vs. 19.79 pg/mL, p=0.030), though IP-10 levels did not differ between different dosage groups. Female neonates demonstrated no significant difference in TRAIL levels across dosage groups, but a significantly lower IP-10 level was observed in the 2-dose group compared to the 3-dose and 4-dose groups (103.32 vs. 151.35 vs. 182.67 pg/mL, p=0.012). However, within each individual dosage cohort, neonatal gender had no significant influence on TRAIL or IP-10 level.

### 3.6 The analysis of correlation between neutralizing antibody inhibition and TRAIL/IP-10

**Figure 3:**
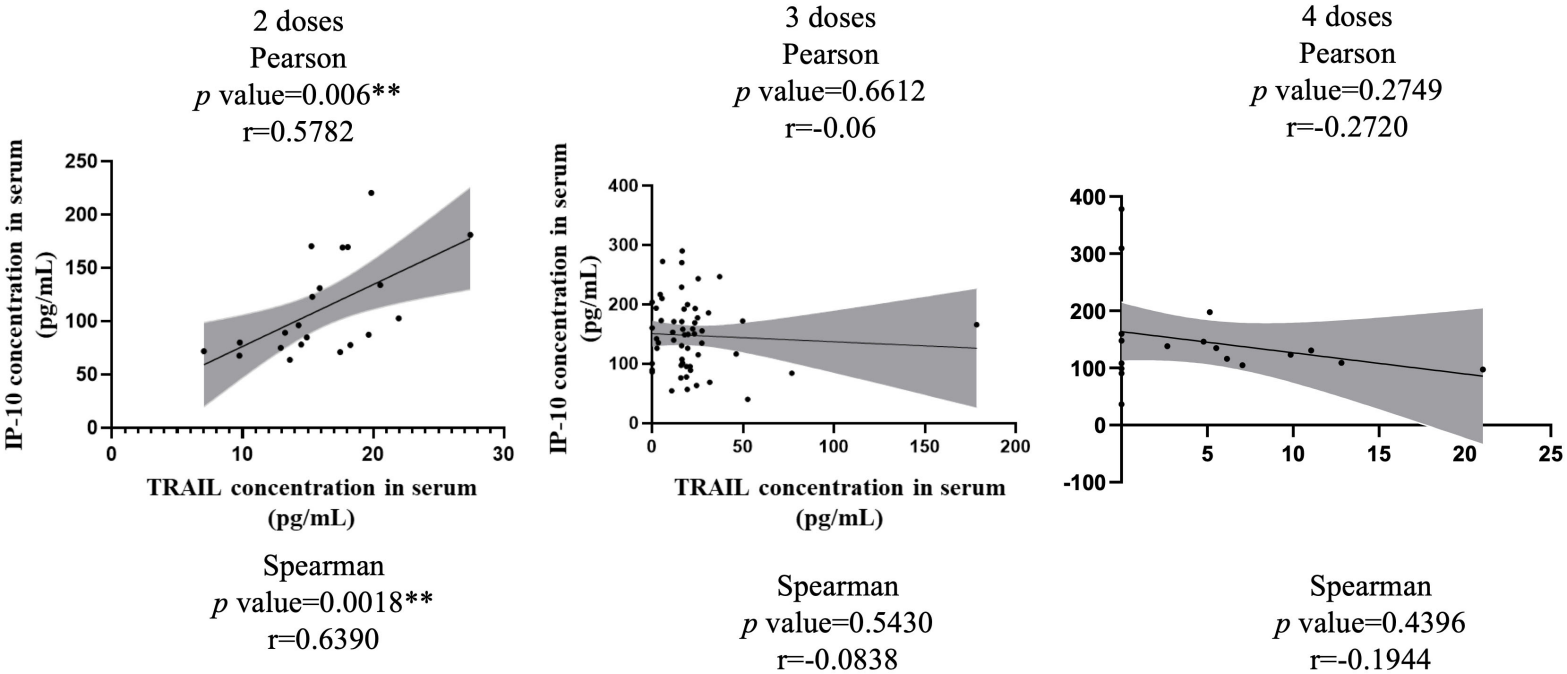
The correlation between levels of TRAIL and IP-10 in maternal blood among participants receiving 2 doses, 3 doses, and 4 doses of COIVD-19 vaccine. TNF-related apoptosis-inducing ligand, TRAIL; interferon gamma-induced protein 10, IP-10. **p-value < 0.01.

**Figure 4:**
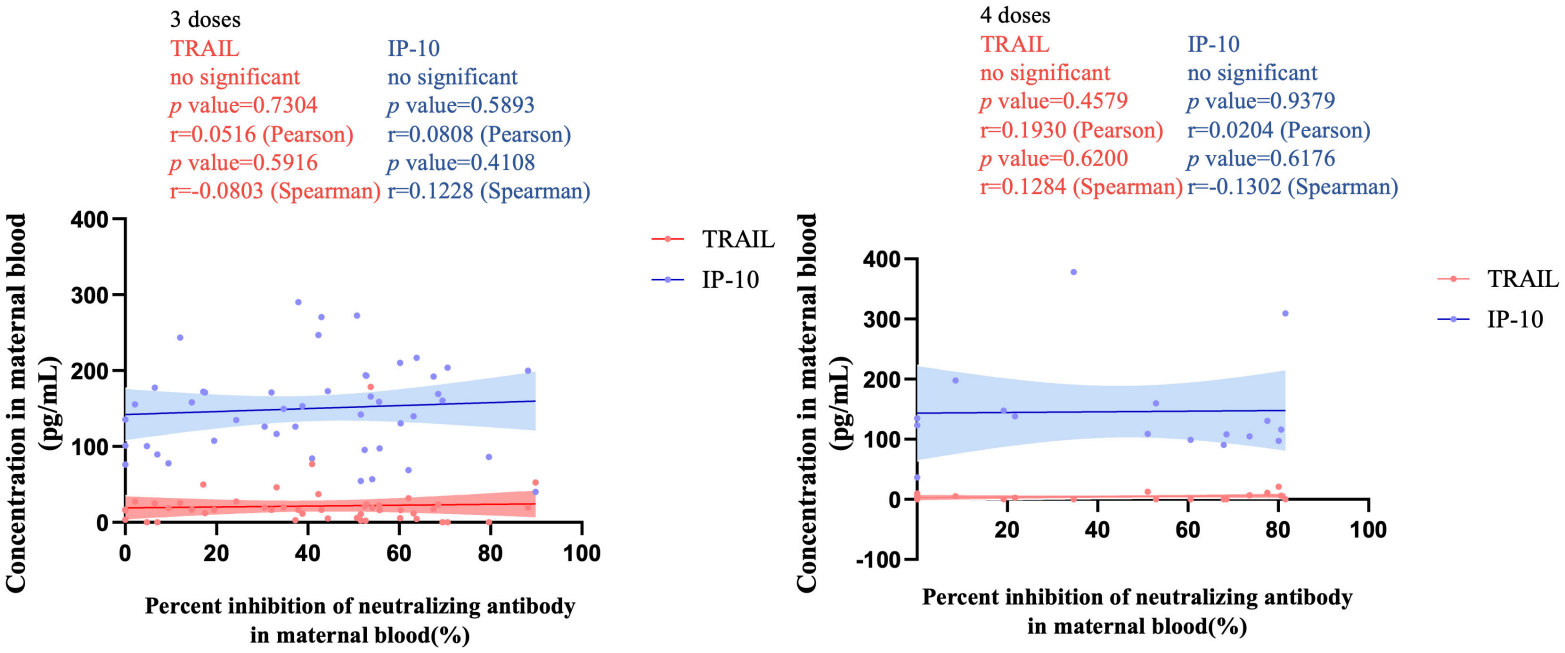
The correlation between TRAIL/IP-10 levels and Nab inhibition rate of omicron type SARS-CoV-2 BA.1 subvariants in maternal blood from participants receiving 3 doses and 4 doses of COIVD-19 vaccine. TNF-related apoptosis-inducing ligand, TRAIL; interferon gamma-induced protein 10, IP-10; Nab, neutralizing antibody.

**Figure 5:**
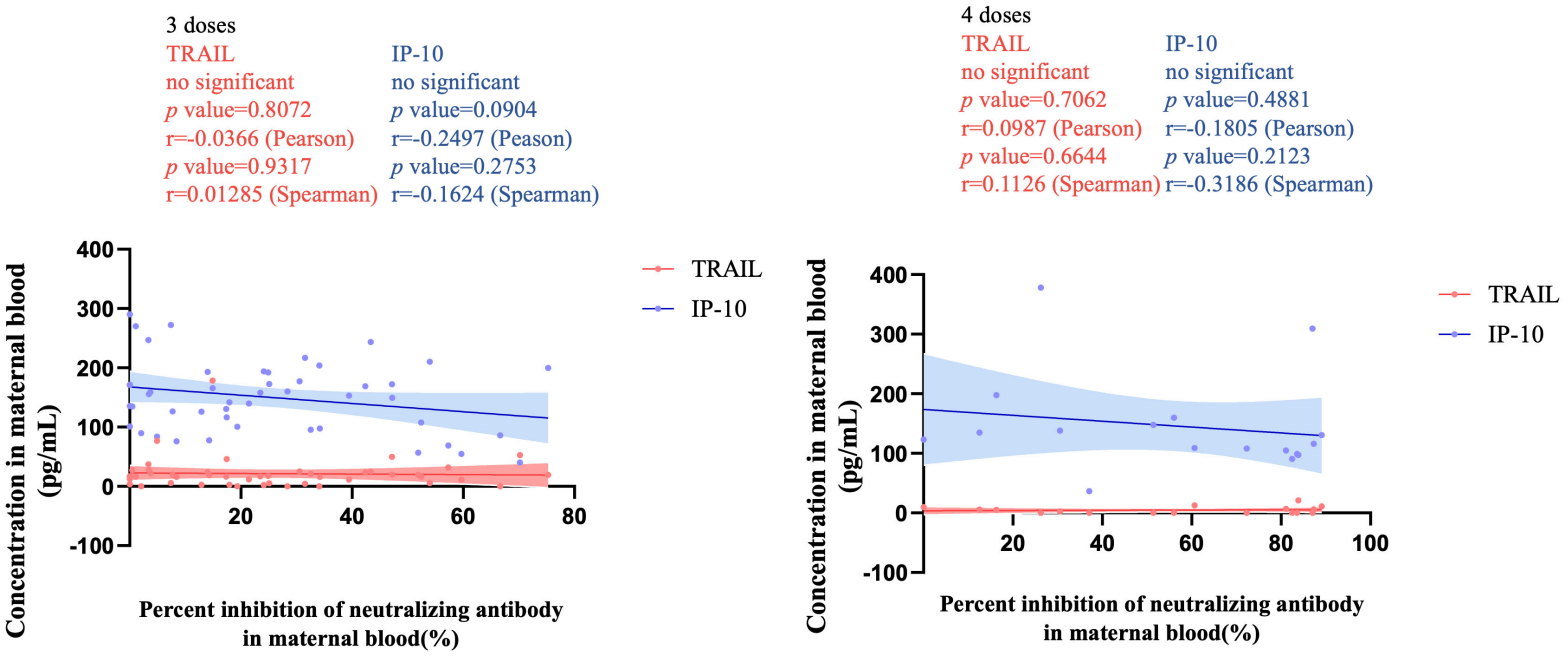
The correlation between TRAIL/IP-10 levels and Nab inhibition rate of omicron type SARS-CoV-2 BA.2 subvariants in maternal blood from participants receiving 3 doses and 4 doses of COIVD-19 vaccine. TNF-related apoptosis-inducing ligand, TRAIL; interferon gamma-induced protein 10, IP-10; Nab, neutralizing antibody.

**Figure 6:**
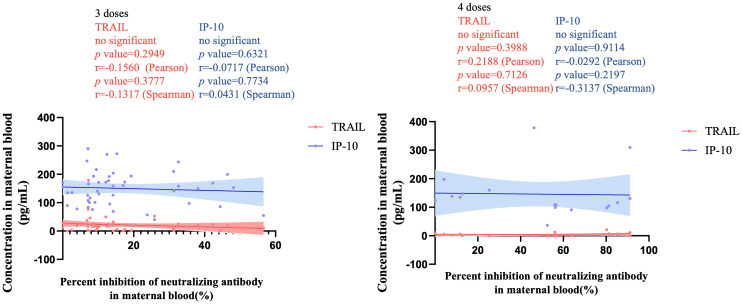
The correlation between TRAIL/IP-10 levels and Nab inhibition rate of omicron type SARS-CoV-2 BA.5 subvariants in maternal blood from participants receiving 3 doses and 4 doses of COIVD-19 vaccine. TNF-related apoptosis-inducing ligand, TRAIL; interferon gamma-induced protein 10, IP-10; Nab, neutralizing antibody.

**Figure 3** illustrates the correlation between TRAIL and IP-10 among different vaccine dosage groups. A positive correlation is evident in the 2-dose cohort between TRAIL and IP-10 (Pearson: p=0.006, r=0.5782; Spearman: p=0.0018, r=0.6390). However, no significant correlation between TRAIL and IP-10 was observed in the 3-dose and 4-dose cohorts. **Figures 4**–**6** present the correlation between the level of TRAIL/IP-10 and Nab inhibition rates in maternal blood against the BA.1, BA.2, and BA.5 SARS-CoV-2 virus subtypes among participants in the 3-dose and 4-dose groups. Within the 3-dose and 4-dose cohorts, no significant differences in TRAIL, IP-10, or Nab inhibition rate were detected in maternal blood samples.

## 4 Discussion

TRAIL is a member of the TNF family, which can trigger extrinsic apoptosis upon binding with receptors containing the intracellular death domain (Sträter and Möller, 2004). As previously mentioned, numerous cells mediating innate immunity enhance the expression of TRAIL following activation by several pro-inflammatory cytokines including IFN-α, IFN-β, IFN-γ, IL-2, and TNF-α (Kashii et al., 1999; Kayagaki et al., 1999a; Takeda et al., 2001; Ehrlich et al., 2003). During viral infections, previous research indicates that IFN expression increases, and that increase results in enhanced expression of TRAIL and its receptor in infected cells. Conversely, normal cells diminish their TRAIL receptor expression. Ultimately, these changes lead to apoptosis of infected cells as a means of controlling infection (Sedger et al., 1999). Experimental evidence has shown that mouse NK cells stimulated with IL-2 and IL-15 can produce TRAIL that can mediate cytotoxicity against various tumor cell lines (Kayagaki et al., 1999a, Kayagaki et al., 1999b; Shepard and Badley, 2009). However, some viruses have evolved mechanisms to activate the TRAIL system, such as inducing NF-κB, allowing the virus to produce proteins that sensitize cells to TRAIL-mediated apoptosis (Wurzer et al., 2004). Certain viral infections can cause uninfected CD4+ and CD8+ lymphocytes to increase the expression of TRAIL and its receptors, which is subsequently accompanied by a significant reduction in lymphocytes (Roe et al., 2004; Gupta et al., 2007). TRAIL induced apoptosis in normal dendritic cells, monocytes, and T-cells is considered a form of immunomodulation (Martínez-Lorenzo et al., 1998; Griffith et al., 1999; Secchiero et al., 2001). In the early stages of infection, TRAIL appears to play a role in suppressing the immune response rather than eliminating the virus or infected cells. However, as the infection progresses, TRAIL may help regulate the removal of infected cells and restrict viral replication, as seen in models of influenza or myocarditis, aiding in infection control (Sato et al., 2001; Brincks et al., 2008). Consequently, lower levels of soluble TRAIL in the blood are linked to septic shock and related mortality (Tian et al., 2013; Schenck et al., 2019). Additionally, in COVID-19 cases, reduced TRAIL levels in blood have been associated with more severe disease outcomes, including extended stays in hospitals and intensive care units (ICU) (Tegethoff et al., 2022).

In pregnant women, TRAIL and its receptors are present in peripheral blood, gestational membranes, and amniotic fluid. Research indicates that the concentration of TRAIL is higher during childbirth, irrespective of labor onset, when compared to preterm birth samples (Lonergan et al., 2003). Furthermore, TRAIL plays a role in protecting vascular endothelial cells (Secchiero et al., 2006), and therefore low levels of TRAIL in the blood are associated with cardiovascular diseases (Volpato et al., 2011). Among pregnant women, such low levels might be associated with early preeclampsia or hypertensive disorders of pregnancy (Zhou et al., 2015). Research on the relationship between TRAIL and vaccination, particularly in pregnant women, has been sparse. This study reveals that pregnant women receiving three doses of COVID-19 vaccines showed higher serum TRAIL levels than those who received two doses of vaccine. TRAIL is found in most cells and its expression increases in activated T-cells. Beyond its role in inducing apoptosis by binding to death receptors, TRAIL can also amplify T-cell proliferation and further enhances IFN-r secretion after TCR binding (Chou et al., 2001). Vaccination with two doses of vaccine can lead to a robust T cell response, which has been observed among patients receiving AZD1222 or BNT162b2 vaccines (Swanson et al., 2021; Gao et al., 2023). Hence, with more doses, the co-stimulation effect of T-cell and TRAIL might be even more potent. Interestingly, this effect diminished with the fourth dose vaccination in our study, which may indicate a robust vaccine memory effect that doesn’t require as much co-stimulation for enhanced vaccine protection.

Our study indicated that the AZD1222 vaccine tends to produce lower TRAIL levels than mRNA-based COVID-19 vaccines. Previous research has demonstrated that individuals primarily vaccinated with AZD1222, when compared with subsequent doses of either AZD1222 or BNT162b2, displayed enhanced immunity triggered by the primary vaccination, but BNT162b2 elicited significantly higher frequencies of spike protein-specific CD4+ and CD8+ T-cells (Barros-Martins et al., 2021). Other research also suggests lower T-cell responses among patients receiving two doses of AZD1222 compared to a those receiving a combination of AZD1222/BNT162b2 or two doses of BNT162b2 (Bánki et al., 2022), which might affect TRAIL levels. In the four-dose group, where 11 out of 17 participants received AZ, the reduction in TRAIL levels could be linked to the dominant use of AZD1222. However, the interval between the last dose and delivery didn’t significantly affect the TRAIL levels. Given that all of our participants were vaccinated during their pregnancy, this suggests that the interval does not notably influence the vaccine’s memory response.

IP-10, also known as CXCL10, is a chemokine that’s rapidly and transiently induced following vaccination and various viral infections (Huang et al., 2005; Sobolev et al., 2016). This chemokine has been shown to trigger the migration, recruitment and activation of monocytes, natural killer (NK) cells, and T-cells to the infection-associated tissue damage site (Singh et al., 2008; Lu et al., 2011). Such chemotactic factors are critical for eliciting protective immune responses at the site of infection (Ramakrishnan, 2012). Elevated serum levels of IP-10 have been correlated with severity of infection or sepsis (Azzurri et al., 2005). Generally, IP-10 is transiently induced by type I or type II interferons and produced by dendritic cells and helper T cells (Sobolev et al., 2016). The gene for the IP-10 chemokine receptor, located in virus-susceptible regions, can be regulated by other proteins, such as CD26 (Casrouge et al., 2012). Therefore, there are multiple mechanisms contributing to increased IP-10 levels during viral infections. Severe COVID-19 has been associated with T-cell apoptosis. T-cell death that has been correlated with IP-10 level (André et al., 2022). In COVID-19 patients, IP-10 levels were observed to be significantly elevated compared to healthy individuals (667.5 vs 127 pg/mL, P<0.001). Moreover, IP-10 levels were positively correlated with disease severity and stood as an independent predictor of ICU mortality (Tegethoff et al., 2022). Previous research also reported that serum levels of IP-10 and MCP-1 were identified as biomarkers of critical illness in COVID-19 patients and tended to increase with disease severity (Chen et al., 2020). Another study also highlighted the association of MCP-3/IP-10 levels with the progression of COVID-19 disease (Yang et al., 2020).

Previous literature has shown that expression of IP-10 and CXCR3 in the decidua of pregnant women is higher than expression in the endometrial tissue of non-pregnant women (Jiang et al., 2021). Additionally, this study also found that mice prone to miscarriage exhibited lower IP-10 levels in their decidua than mice with normal pregnancies (Jiang et al., 2021). Therefore, IP-10 can shape a pro-inflammatory immune microenvironment during early pregnancy via the distribution of immune cells and the generation of pro-inflammatory cytokines (Jiang et al., 2021). Women with preeclampsia, which may be an anti-angiogenic state with augmented systemic inflammatory response, also displayed significantly elevated serum concentrations of IP-10 compared to women with normal pregnancies (Gotsch et al., 2007b). Similarly, elevated IP-10 levels can be detected in the maternal blood of pregnant women with acute pyelonephritis (Gotsch et al., 2007a).

Previous research had revealed an increase in IP-10 levels after vaccinations (Tang et al., 2023), and the increase was evident in healthy individuals as well as in elderly or cancer patients after administration of the BNT162b2 vaccine (Konnova et al., 2022). According to the literature, after receiving a second vaccine dose, a memory response was induced, leading to a significant increase in levels of IFN-g, IP-10/CXCL10, IL-6, and TNF-a that were crucial for quickly recruiting and activating effector immune cells (Bergamaschi et al., 2021). IP-10 is commonly released by various cells including leukocytes, neutrophils, eosinophils, monocytes, and mesenchymal cells during inflammation, and it can facilitate the chemotaxis of CXCR3+ cells that are predominantly activated T and B lymphocytes (Loetscher et al., 1998). Our recent findings demonstrated a higher IP-10 level after three doses of COVID-19 vaccines was comparable to IP-10 level following receipt of two doses of vaccine. By the fourth dose, the body’s memory response appears to be sufficiently robust. It is possible that this reduces the need for the involvement of IP-10 compared to the 2- or 3-dose cohorts. Additionally, patients that received AZD1222 vaccine as the primary vaccination demonstrated elevated IP-10 levels. While the underlying reason remains elusive, it might be linked to the adenovirus vector of AZD1222 vaccines, which can concurrently invoke an inflammatory response and therefore increase IP-10 (Azzarone et al., 2021; Ostrowski et al., 2021). In the two-dose group, AZD1222 was not used. However, in the three-dose group, 18 out of 54 subjects received AZD1222, and in the four-dose group, 11 out of 17 did. This distribution could influence the observed values.

Our previous research reported that 2-dose vaccinations produced greater Nab inhibition for various strains of SARS-CoV-2 including wildtype, alpha, beta, gamma, and delta type, than 1-dose vaccinations according to values observed in maternal blood and corresponding neonatal cord blood (Shen et al., 2022; Chen et al., 2022a). Other research reported in the literature has indicated that booster COVID-19 vaccination can produce a more potent Nab response against the Omicron SARS-CoV-2 variants including several novel subvariants (Lin et al., 2023). This enhanced Nab protection from booster vaccines can also be observed in maternal blood and neonatal cord blood (Munoz et al., 2023). The above findings are compatible with those of our current study indicating that the four-dose group exhibited higher Nab inhibition against the BA.1, BA.2, and BA.5 Omicron variant strains compared to the three-dose group. It also aligns with current trend indicating that receiving a fourth dose not only strengthens the vaccine’s memory response and protection against Omicron variants but also amplifies Nab protection, especially in pregnant women.

In this study, TRAIL and IP-10 levels varied in the maternal serum of study participants receiving different doses of COVID-19 vaccine. However, when assessing Nab inhibition, it appears that the levels of TRAIL and IP-10 had no significant impact. The reasons for this remain unclear, but it is possible that TRAIL and IP-10 may be involved in the body’s memory response following vaccination, in modulating post-vaccination inflammatory reactions, and in regulating the capacity for antibody production. With increased vaccine doses, the memory response of immunity may be enhanced, and the body’s inflammatory reaction further intensified, which could lead to alterations in the levels of TRAIL and IP-10. Nevertheless, in our study, Nab inhibition remained consistently high and even increased with subsequent doses of COVID-19 vaccine.

In our current study, subjects receiving two doses of COVID-19 vaccine demonstrated a consistent positive correlation with TRAIL and IP-10 level. This may be due to the fact that the vaccine doses were mRNA-based COVID-19 vaccines. However, as the vaccine dosage number increased to three or four, the vaccine’s memory response might have intensified. Moreover, the complexity in vaccine composition arises as some participants had been administered AZD1222 vaccine. The adenovirus vector of AZD1222 vaccine may also trigger inflammatory reactions that may potentially influence the post-vaccination inflammatory states and other related reactions (Azzarone et al., 2021; Ostrowski et al., 2021). Therefore, various factors might disrupt and affect the correlation between the levels of TRAIL and IP-10, and therefore no significant correlation can be obviously detected for the 3-doses and 4-doses cohorts.

To the best of our knowledge, this is the first study to investigate the variations of TRAIL and IP-10 in the blood of pregnant women following COVID-19 vaccination. Our study also had limitations. First, there was discrepancy in the distribution among the participant groups receiving different doses of vaccines. When comparing the groups, the sample size was uneven: the 2-dose group had 21 participants, the 4-dose group had 17, and the 3-dose group contained 55 subjects. Additionally, the vaccination combinations were inconsistent for the participants in our study. While the two-dose group did not receive AZD1222 vaccine, some participants in the three-dose and four-dose groups did. The above discrepancy of vaccine combination could also affect our findings when making comparisons among the vaccine dose groups. Enrolling a greater number of participants in a future study is advised and could validate our findings.

## 5 Conclusions

The levels of TRAIL and IP-10 increase as the number of vaccine doses increases. However, upon reaching the fourth dose, TRAIL levels decrease while IP-10 levels rise. Furthermore, those who have previously received the AZD1222 vaccine tended to exhibit lower TRAIL levels and higher IP-10 levels. Despite these variations, a higher number of vaccine doses consistently lead to enhanced Nab inhibition, which appears to be independent of TRAIL and IP-10 levels. The fluctuations in TRAIL and IP-10 levels upon vaccination might serve as a reflection of the body’s memory response to the vaccine. Further research is needed with more participants.

## Data availability statement

The original contributions presented in the study are included in the article/**Supplementary Material**. Further inquiries can be directed to the corresponding author.

## Ethics statement

The studies involving humans were approved by Kaohsiung Medical University Hospital (IRB No. KMUHIRB-SV(II)-20210087, an ethics review committee, on August 7th, 2021). The studies were conducted in accordance with the local legislation and institutional requirements. The participants provided their written informed consent to participate in this study.

## Author contributions

W-CC: Conceptualization, Data curation, Formal analysis, Funding acquisition, Investigation, Methodology, Project administration, Software, Validation, Visualization, Writing – original draft, Writing – review & editing. S-YH: Data curation, Investigation, Methodology, Software, Validation, Visualization, Writing – original draft. C-FS: Data curation, Methodology, Resources, Supervision, Writing – original draft. H-YC: Investigation, Resources, Writing – original draft. C-RK: Investigation, Resources, Writing – original draft. D-JS: Investigation, Resources, Writing – original draft. C-JS: Conceptualization, Investigation, Methodology, Project administration, Supervision, Writing – review & editing. C-MC: Conceptualization, Data curation, Formal analysis, Funding acquisition, Investigation, Methodology, Project administration, Resources, Supervision, Validation, Visualization, Writing – original draft, Writing – review & editing.

## Supplementary material

Supplementary Figure 1 The dot blots of TRAIL and IP-10 among different vaccine doses groups. TNF-related apoptosis-inducing ligand, TRAIL; Interferon gamma-induced protein 10, IP-10.

Supplementary Figure 2 Neutralizing antibody (Nab) inhibition rates to omicron type SARS-CoV-2 BA.1, BA.2, and BA.5 subvariants in maternal blood from participants receiving 3 and 4 doses of COVID-19 vaccine.

Supplementary Figure 3 TRAIL concentrations in maternal blood among different regimens of Tdap (tetanus toxoid, reduced diphtheria toxoid, and acellular pertussis vaccines)/Flu (influenza) vaccination during pregnancy from participants receiving 3 doses of mRNA-based COVID-19 vaccine. TNF-related apoptosis-inducing ligand, TRAIL.

Supplementary Figure 4 IP-10 concentrations in maternal blood among different regimens of Tdap (tetanus toxoid, reduced diphtheria toxoid, and acellular pertussis vaccines)/Flu (influenza) vaccination during pregnancy from participants receiving 3 doses of mRNA-based COVID-19 vaccine. Interferon gamma-induced protein 10, IP-10.

Supplementary Figure 5 Neutralizing antibody (Nab) inhibition rates to omicron type SARS-CoV-2 BA.1, BA.2, and BA.5 subvariants in maternal blood among different intervals between last COVID-19 vaccine dose to childbirth from participants receiving 3 doses of COVID-19 vaccine.

Supplementary Figure 6 Neutralizing antibody (Nab) inhibition rates to omicron type SARS-CoV-2 BA.1, BA.2, and BA.5 subvariants in maternal blood among different regimens of Tdap (tetanus toxoid, reduced diphtheria toxoid, and acellular pertussis vaccines)/Flu (influenza) vaccination during pregnancy from participants receiving 3 doses of mRNA-based COVID-19 vaccine.
