# Supplementary material for: TRAIL and IP-10 dynamics in pregnant women post COVID-19 vaccination: associations with neutralizing antibody potency
Source: Front Cell Infect Microbiol. 2024 Mar 20;14:1358967. doi: 10.3389/fcimb.2024.1358967 (PMC10987851; doi:10.3389/fcimb.2024.1358967)
Supplement: Supplementary file 1 [file Table_1.docx]

**Table S1.** TRAIL and IP-10 levels of maternal blood in those with or without previous AZD1222 vaccination among participants receiving 2, 3 and 4 doses of COVID-19 vaccine.

|  | **N** | **TRAIL (pg/mL)** | **IP-10 (pg/mL)** |
| --- | --- | --- | --- |
| 2 doses  With AZ  Without AZ  p value | 0  21 | NA  16.07  NA | NA  111.49  NA |
| 3 doses  With AZ  Without AZ  p value | 19  36 | 6.68  29.48  0.013 | 162.99  139.91  0.169 |
| 4 doses  With AZ  Without AZ  p value | 11  6 | 3.38  7.35  0.207 | 173.83  94.76  0.056 |
| Overall cohorts  With AZ  Without AZ  p value | 30  63 | 5.47  22.90  0.001 | 166.97  126.14  0.0028 |

NA, not available; TNF-related apoptosis-inducing ligand, TRAIL; interferon gamma-induced protein 10, IP-10; AZ, AZD1222 vaccine.

**Table S2.** TRAIL and IP-10 levels of maternal blood in those with different intervals between last COVID-19 vaccine dose to childbirth among participants receiving 2, 3, and 4 doses of COVID-19 vaccine.

|  | **N** | **TRAIL (pg/mL)** | **IP-10 (pg/mL)** |
| --- | --- | --- | --- |
| 2 doses  0-4 weeks  5-8 weeks  9-12 weeks  p value | 7  7  7 | 16.39  15.79  16.03  0.972 | 133.94  108.09  92.43  0.240 |
| 3 doses  0-4 weeks  5-8 weeks  9-12 weeks  p value | 19  21  15 | 16.49  17.15  34.33  0.080 | 154.68  145.37  142.81  0.823 |
| 4 doses  0-4 weeks  5-8 weeks  9-12 weeks  > 12 weeks  p value | 6  6  2  3 | 6.10  4.68  0  5.52  0.707 | 140.59  182.77  119.12  100.77  0.549 |
| Overall cohorts  0-4 weeks  5-8 weeks  9-12 weeks  > 12 weeks  p value | 32  34  24  3 | 14.52  14.67  26.13  5.52  0.099 | 147.50  144.29  126.14  100.77  0.402 |

TNF-related apoptosis-inducing ligand, TRAIL; interferon gamma-induced protein 10, IP-10

**Table S3.** TRAIL and IP-10 levels of maternal blood in those with different maternal age among participants receiving 2, 3, and 4 doses of COVID-19 vaccine.

|  | **N** | | **TRAIL (pg/mL)** | | **IP-10 (pg/mL)** | |
| --- | --- | --- | --- | --- | --- | --- |
| 2 doses  Age <30  Age 30-35  Age 35-40  Age >40  p value | 3  7  6  5 | | 12.55  13.57  19.71  17.32  0.029 | | 79.83  95.56  124.19  137.54  0.232 | |
| 3 doses  Age <30  Age 30-35  Age 35-40  Age >40  p value | 13  19  17  6 | | 34.71  17.55  14.78  25.39  0.163 | | 158.73  142.47  143.02  155.37  0.854 | |
| 4 doses  Age <30  Age 30-35  Age 35-40  Age >40  p value | 3  8  5  1 | | 2.35  5.79  5.57  0  0.742 | | 77.33  177.82  145.41  99.11  0.333 | |
| Overall cohorts  Age <30  Age 30-35  Age 35-40  Age >40  p value | 19  34  28  12 | 26.10  13.97  14.20  19.92  0.175 | | 133.42  141.13  139.41  143.26  0.971 | |  |

TNF-related apoptosis-inducing ligand, TRAIL; interferon gamma-induced protein 10, IP-10

**Table S4.** TRAIL and IP-10 levels of maternal blood in those with different maternal BMI among participants receiving 2, 3, and 4 doses of COVID-19 vaccine.

|  | **N** | **TRAIL (pg/mL)** | **IP-10 (pg/mL)** |
| --- | --- | --- | --- |
| 2 doses  BMI <25  BMI 25-30  BMI >30  p value | 6  9  6 | 15.49  15.63  17.30  0.754 | 109.51  113.31  110.73  0.988 |
| 3 doses  BMI <25  BMI 25-30  BMI >30  p value | 17  19  19 | 21.46  26.25  17.09  0.560 | 135.78  167.48  139.13  0.200 |
| 4 doses  BMI <25  BMI 25-30  BMI >30  p value | 6  10  1 | 5.15  5.04  0  0.744 | 100.55  171.76  159.77  0.258 |
| Overall cohorts  BMI <25  BMI 25-30  BMI >30  p value | 29  38  26 | 16.86  18.15  16.48  0.946 | 123.06  155.78  133.37  0.088 |

TNF-related apoptosis-inducing ligand, TRAIL; interferon gamma-induced protein 10, IP-10; BMI, body mass index.

**Table S5.** TRAIL and IP-10 levels of maternal blood in those with different regimens of Tdap/Flu vaccinations during pregnancy among participants receiving 3 and 4 doses of COVID-19 vaccine.

|  | **N** | **TRAIL (pg/mL)** | **IP-10 (pg/mL)** |
| --- | --- | --- | --- |
| 3 doses  Tdap only  Flu only  Tdap + Flu  No Tdap/Flu  p value | 28  1  16  10 | 22.40  49.88  18.23  21.95  0.695 | 146.89  172.22  157.63  133.14  0.752 |
| 4 doses  Tdap only  Tdap + Flu  No Tdap/Flu  p value | 3  11  3 | 0  5.98  5.16  0.337 | 118.29  162.58  112.49  0.560 |
| Overall cohorts  Tdap only  Flu only  Tdap + Flu  No Tdap/Flu  p value | 31  1  27  34 | 20.24  49.88  13.24  16.84  0.263 | 143.97  172.22  159.64  117.95  0.061 |

TNF-related apoptosis-inducing ligand, TRAIL; interferon gamma-induced protein 10, IP-10; Tdap, tetanus toxoid, reduced diphtheria toxoid, and acellular pertussis vaccines; Flu, influenza vaccine.

**Table S6.** TRAIL and IP-10 levels of maternal blood in those with different neonatal body weight among participants receiving 2, 3, and 4 doses of COVID-19 vaccine.

|  | **N** | **TRAIL (pg/mL)** | **IP-10 (pg/mL)** |
| --- | --- | --- | --- |
| 2 doses  < 2500 gm  2500-3000  3000-3500  > 3500 gm  p value | 1  10  7  2 | 7.04  15.05  16.94  20.80  0.068 | 71.74  102.79  118.79  94.89  0.669 |
| 3 doses  < 2500 gm  2500-3000  3000-3500  > 3500 gm  p value | 1  18  29  7 | 17.94  28.64  16.58  24.85  0.476 | 148.86  133.16  155.90  152.46  0.648 |
| 4 doses  < 2500 gm  2500-3000  3000-3500  > 3500 gm  p value | 1  8  5  3 | 0  5.11  4.41  6.11  0.874 | 108.11  134.83  180.52  130.45  0.759 |
| Overall cohorts  < 2500 gm  2500-3000  3000-3500  > 3500 gm  p value | 3  36  41  12 | 8.33  19.64  15.16  19.49  0.684 | 109.57  125.09  152.56  137.36  0.222 |

TNF-related apoptosis-inducing ligand, TRAIL; interferon gamma-induced protein 10, IP-10; BW, body weight.

**Table S7.** TRAIL and IP-10 levels of maternal blood in those with different neonatal gender among participants receiving 2, 3, and 4 doses of COVID-19 vaccine.

|  | **N** | **TRAIL (pg/mL)** | **IP-10 (pg/mL)** |
| --- | --- | --- | --- |
| 2 doses  Male baby  Female baby  p value | 6  15 | 17.77  15.39  0.293 | 131.91  103.32  0.353 |
| 3 doses  Male baby  Female baby  p value | 31  24 | 19.79  23.94  0.561 | 145.21  151.35  0.705 |
| 4 doses  Male baby  Female baby  p value | 9  8 | 5.30  4.19  0.721 | 113.26  182.67  0.109 |
| Overall cohorts  Male baby  Female baby  p value | 46  47 | 16.69  17.85  0.793 | 137.22  141.35  0.752 |

TNF-related apoptosis-inducing ligand, TRAIL; interferon gamma-induced protein 10, IP-10.
