# Supplementary figures and images for: TRAIL and IP-10 dynamics in pregnant women post COVID-19 vaccination: associations with neutralizing antibody potency
Source: Front Cell Infect Microbiol. 2024 Mar 20;14:1358967. doi: 10.3389/fcimb.2024.1358967 (PMC10987851; doi:10.3389/fcimb.2024.1358967)

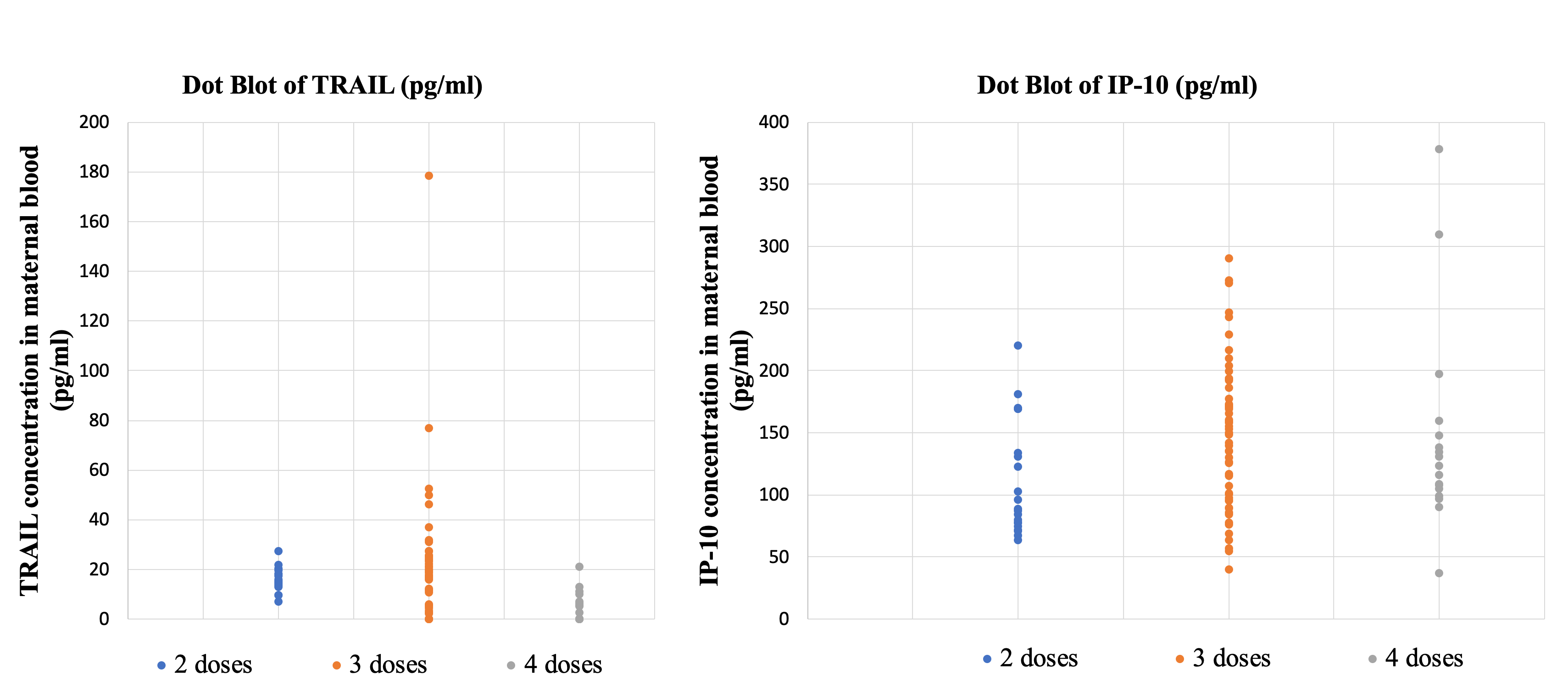

Supplement: Supplementary Figure 1 — The dot blots of TRAIL and IP-10 among different vaccine doses groups. TNF-related apoptosis-inducing ligand, TRAIL; Interferon gamma-induced protein 10, IP-10. [file Image_1.tiff]

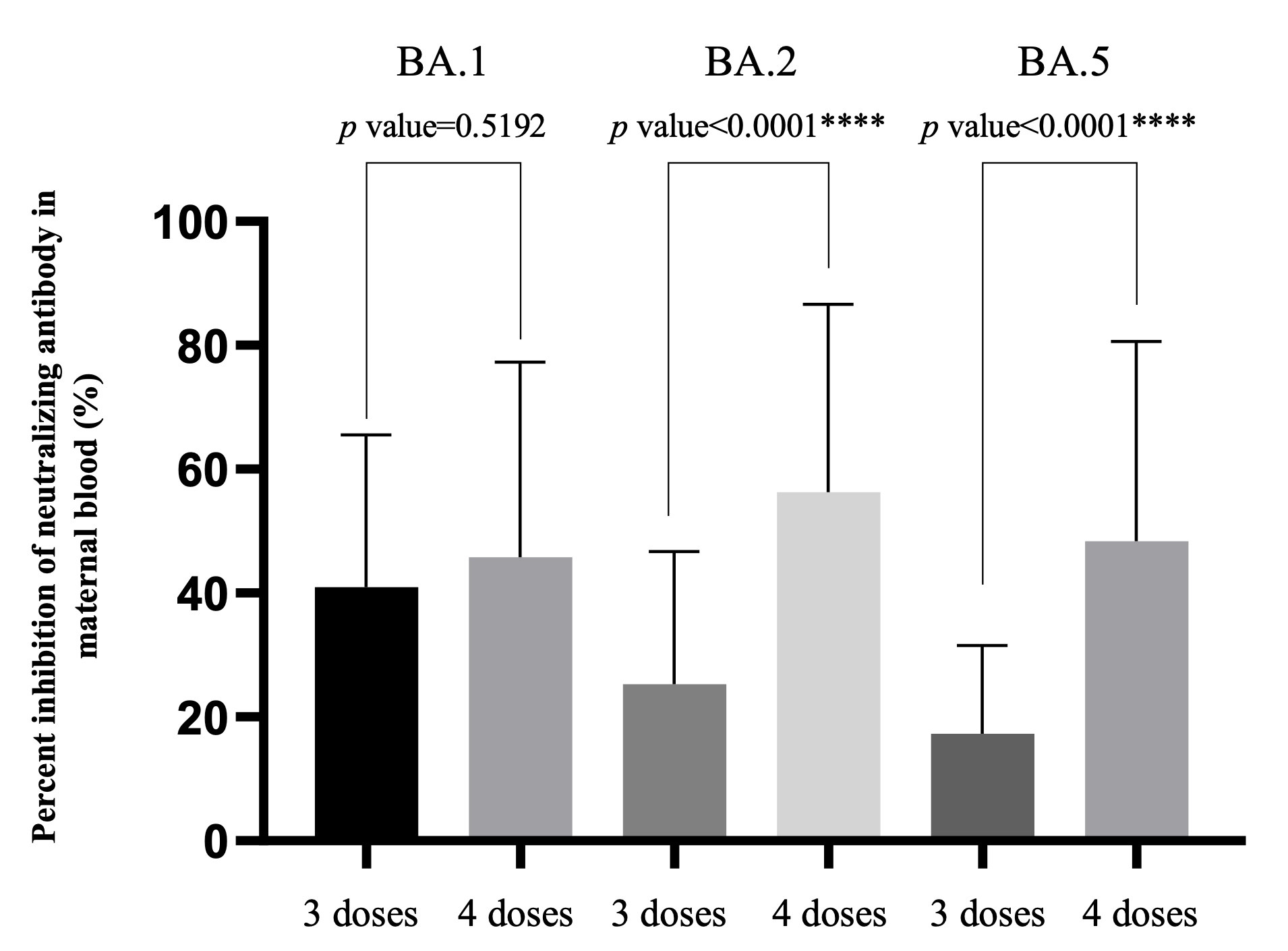

Supplement: Supplementary Figure 2 — Neutralizing antibody (Nab) inhibition rates to omicron type SARS-CoV-2 BA.1, BA.2, and BA.5 subvariants in maternal blood from participants receiving 3 and 4 doses of COVID-19 vaccine. [file Image_2.tif]

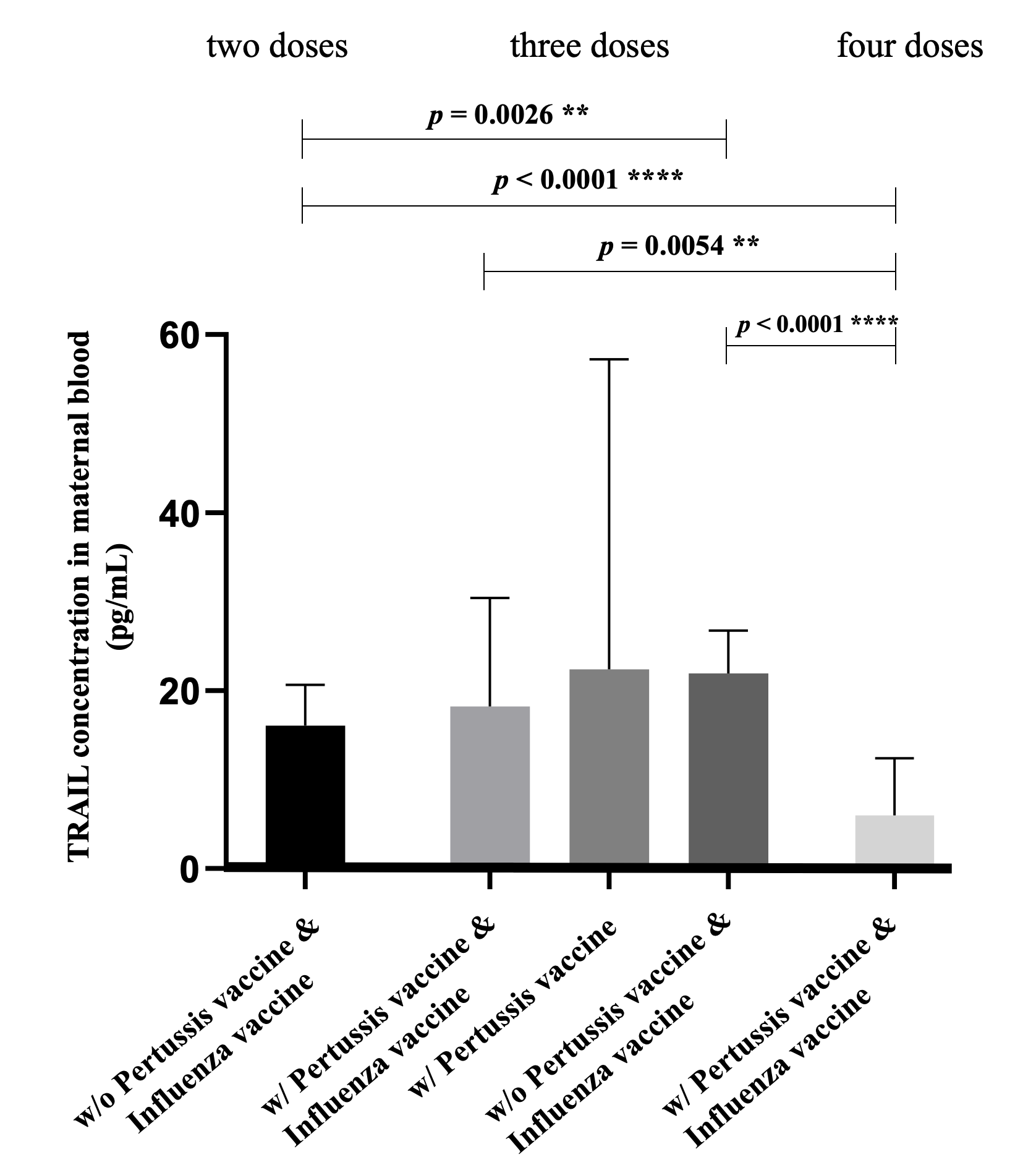

Supplement: Supplementary Figure 3 — TRAIL concentrations in maternal blood among different regimens of Tdap (tetanus toxoid, reduced diphtheria toxoid, and acellular pertussis vaccines)/Flu (influenza) vaccination during pregnancy from participants receiving 3 doses of mRNA-based COVID-19 vaccine. TNF-related apoptosis-inducing ligand, TRAIL. [file Image_3.tiff]

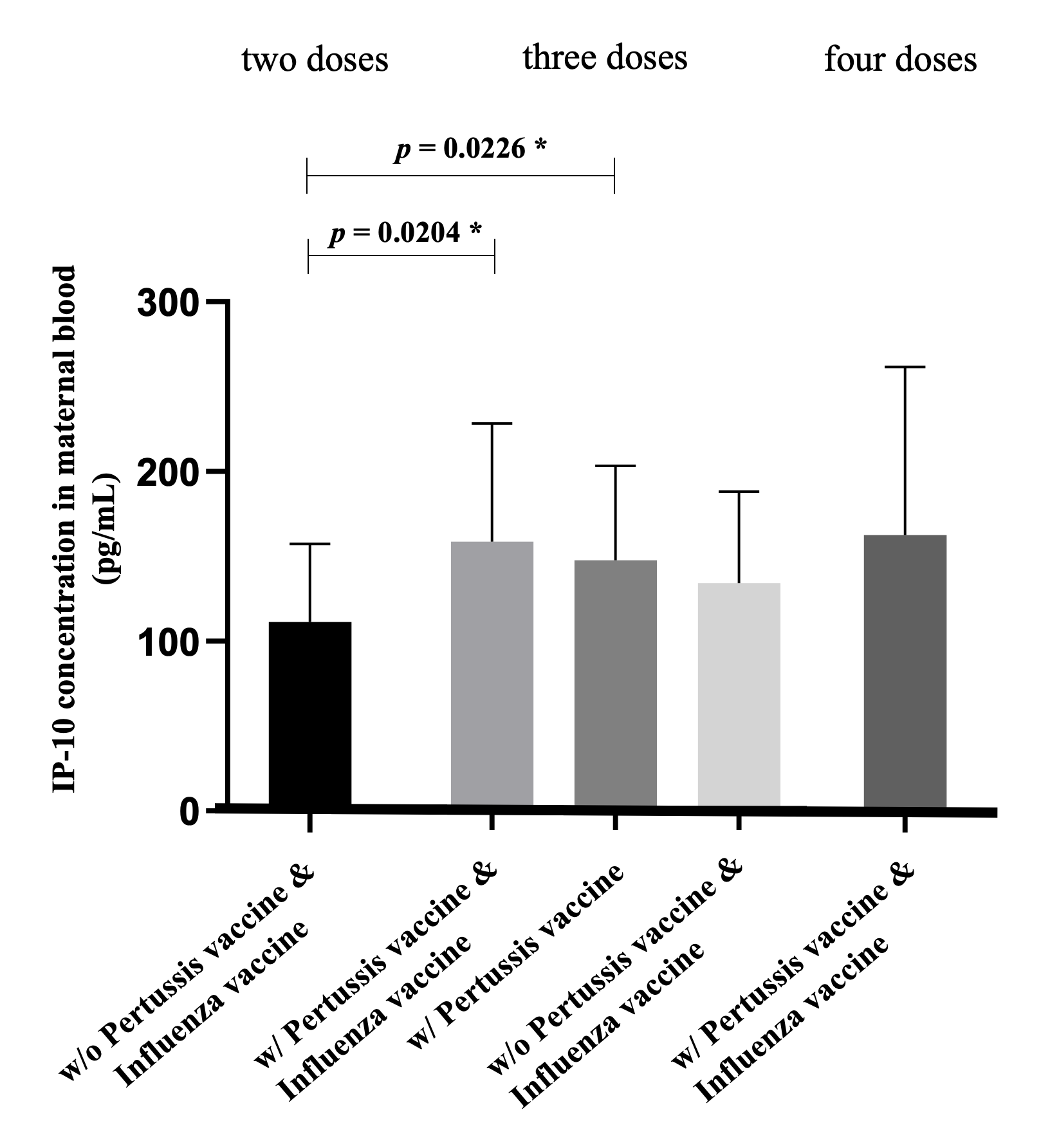

Supplement: Supplementary Figure 4 — IP-10 concentrations in maternal blood among different regimens of Tdap (tetanus toxoid, reduced diphtheria toxoid, and acellular pertussis vaccines)/Flu (influenza) vaccination during pregnancy from participants receiving 3 doses of mRNA-based COVID-19 vaccine. Interferon gamma-induced protein 10, IP-10. [file Image_4.tiff]

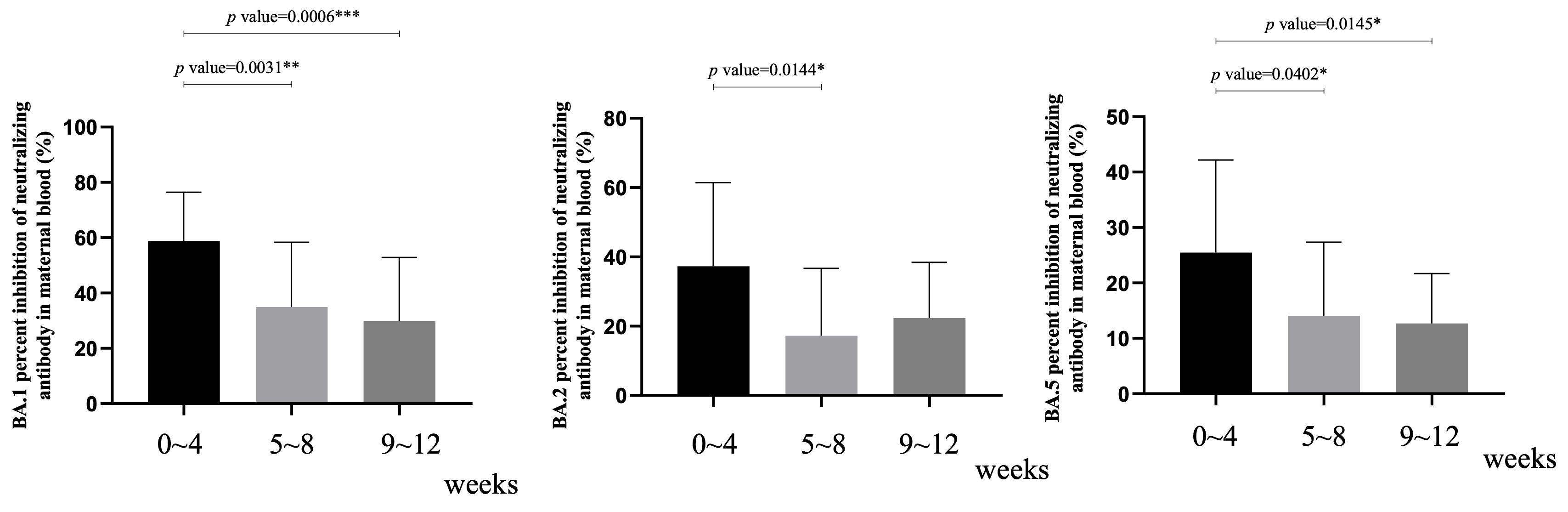

Supplement: Supplementary Figure 5 — Neutralizing antibody (Nab) inhibition rates to omicron type SARS-CoV-2 BA.1, BA.2, and BA.5 subvariants in maternal blood among different intervals between last COVID-19 vaccine dose to childbirth from participants receiving 3 doses of COVID-19 vaccine. [file Image_5.tif]

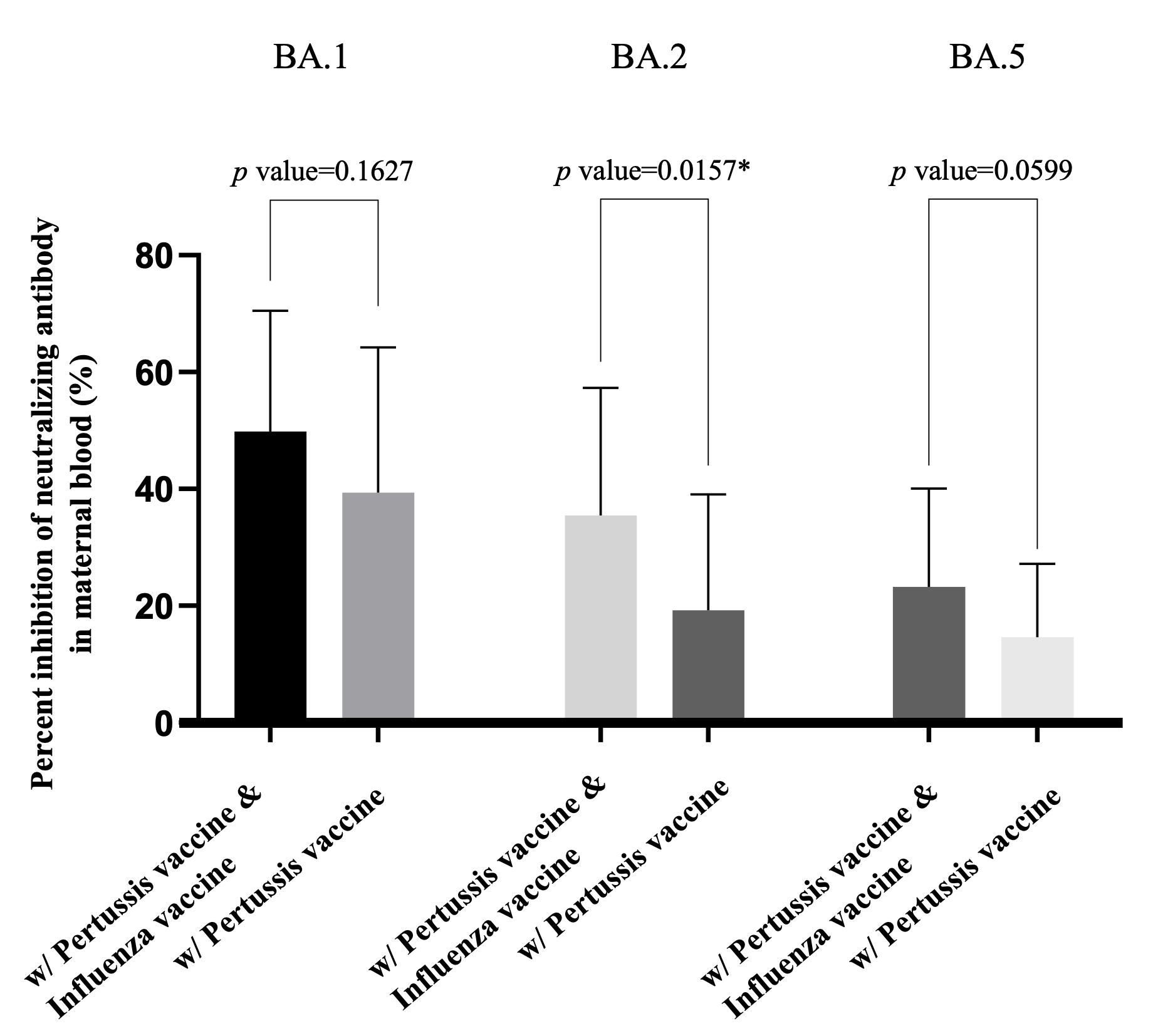

Supplement: Supplementary Figure 6 — Neutralizing antibody (Nab) inhibition rates to omicron type SARS-CoV-2 BA.1, BA.2, and BA.5 subvariants in maternal blood among different regimens of Tdap (tetanus toxoid, reduced diphtheria toxoid, and acellular pertussis vaccines)/Flu (influenza) vaccination during pregnancy from participants receiving 3 doses of mRNA-based COVID-19 vaccine. [file Image_6.tif]
